# A new roadmap for an age-inclusive workforce management practice and an international policies comparison

**DOI:** 10.12688/openreseurope.17159.1

**Published:** 2024-04-24

**Authors:** Niloofar Katiraee, Nicola Berti, Ajay Das, Ilenia Zennaro, Riccardo Aldrighetti, Vlado Dimovski, Darja Peljhan, Debra Dobbs, Christoph Glock, Gail Pacheco, Patrick Neumann, Ami Ogawa, Daria Battini

**Affiliations:** 1 Department of Management and Engineering, Universita degli Studi di Padova, Vicenza, Italy; 2 Narendra Paul Loomba Department of Management, Baruch College, New York, USA; 3 School of Economics and Business, University of Ljubljana, Ljubljana, Slovenia; 4 School of Aging Studies, University of South Florida, Tampa, USA; 5 Institute of Production and Supply Chain Management, Technische Universitat Darmstadt, Darmstadt, Germany; 6 NZ Work Research Institute, Auckland University of Technology, Auckland, New Zealand; 7 Department of Mechanical and Industrial Engineering, Toronto Metropolitan University, Toronto, Canada; 8 Department of System Design Engineering, Faculty of Science and Technology, Keio University, Minato, Japan

**Keywords:** Ageing workforce management, age-friendly policy, ageing workforce practices, international comparisons, industry 5.0, social responsibility, social sustainability, inclusive workforce

## Abstract

**Background:**

Worldwide, the worker population age is growing at an increasing rate. Consequently, government institutions and companies are being tasked to find new ways to address age-related workforce management challenges and opportunities. The development of age-friendly working environments to enhance ageing workforce inclusion and diversity has become a current management and national policy imperative. Since an ageing workforce population is a spreading worldwide trend, an identification and analysis of worker age related best practices across different countries would help the development of novel palliative paradigms and initiatives.

**Methods:**

This study proposes a new systematic research-based roadmap that aims to support executives and administrators in implementing an age-inclusive workforce management program. The roadmap integrates and builds on published literature, best practices, and international policies and initiatives that were identified, collected, and analysed by the authors. The roadmap provides a critical comparison of age-inclusive management practices and policies at three different levels of intervention: international, country, and company. Data collection and analysis was conducted simultaneously across eight countries: Canada, France, Germany, Italy, Japan, New Zealand, Slovenia, and the USA.

**Results and conclusions:**

The findings of this research guide the development of a framework and roadmap to help manage the challenges and opportunities of an ageing workforce in moving towards a more sustainable, inclusive, and resilient labour force.

## Plain language summary

This article presents an in-depth analysis of applied and proposed policies, strategies, and approaches for managing aging workforce in workplaces. The primary objective of this paper is to propose a new systematic research-based roadmap that aims to support executives and administrators in implementing an age-inclusive workforce management program.

The roadmap integrates and builds on published literature, best practices, and international policies and initiatives that were identified, collected, and analysed by the authors.

The roadmap provides a critical comparison of age-inclusive management practices and policies at three different levels of intervention: international, country, and company. Data collection and analysis were conducted simultaneously across the mentioned eight countries: Canada, France, Germany, Italy, Japan, New Zealand, Slovenia, and the USA.

## 1. Introduction

The aging of the global workforce is a prevailing and transformative phenomenon that has far-reaching implications for societies, economies, and organizations around the world. As life expectancies increase and birth rates decline, the proportion and number of older individuals in the workforce are growing steadily. The population of both European and international countries such as Japan, US, Canada, and New Zealand is progressively aging ( OECD, 2022), resulting in a rise in the average retirement age in many Organisations for Economic Cooperation and Development (OECD) member countries ( Calzavara *et al.*, 2020). This demographic shift presents both challenges and opportunities for institutions, governments, and companies.

As the workforce ages, organizations face several challenges related to managing and accommodating the needs of older employees. Hence, the investigation of age-oriented system design and employability of aging workers can provide valuable insights into the costs and benefits of retaining experienced employees versus their retirement ( Bogataj *et al.*, 2019). Furthermore, creating age-friendly workplaces is essential to utilize the expertise and experiences of aging workers effectively, with regard to physical manufacturing environments. Workforce well-being and sustainable development is also central in the recent Industry 5.0 paradigms ( European Commission, 2021) and fits the well-known ESG (Environmental, Social, and Governance) and DEI (Diversity, equity, and inclusion) criteria and investment goals.

At the international level, the newly developed ISO 25550:2022 provides general requirements and guidelines for an age-inclusive workforce designed to keep the workers active and motivated in the workplace for as long as possible ( https://www.iso.org/standard/76420.html). Recent studies investigate how ageing workers characteristics affect the design of inclusive working environments, especially in manufacturing and labour-intensive sectors ( Botti *et al.*, 2017; Calzavara *et al.*, 2020; Katiraee *et al.*, 2019; Katiraee *et al.*, 2020; Katiraee *et al.*, 2021a), while underlining the need for individualized and diversified workplace design ( Neumann *et al.*, 2021 and Sgarbossa *et al.*, 2020). Promoting the inclusion and retention of older workers in the labour market is essential for economic growth and sustainability. With a shrinking pool of younger talent, tapping into the potential of older employees becomes imperative for maintaining workforce continuity, permit efficient knowledge transfer and meeting labour demands in various industries ( OECD, 2020).

This paper provides a new and comprehensive methodological roadmap to support organizations in implementing an age-inclusive workforce management program. The proposed roadmap defines 6 action domains and 3 levels of intervention: international, country/regional and company level. Data was collected by the global partners involved in the MAIA H2020-MSCA-RISE Project ( www.maiaproject.eu), examining and comparing best practices and policies developed in the 8 countries involved in this study (Canada, France, Germany, Italy, Japan, New Zealand, Slovenia, and USA). The outcome provides an overview of the current readiness level of each country involved in the MAIA project to deal with ageing population trends and conducts a data-based benchmark analysis. The research analysis compares the approaches taken by various countries and companies, fostering knowledge sharing and the adoption of best practices to better manage and integrate the aging workforce. Furthermore, the study's findings may help institutions, governments, and companies develop more age-friendly working and living spaces, leading to enhanced diversity management and overall inclusion of older employees in the workforce.

The rest of the paper is organized as follows. Section 2 provides a literature review on ageing workforce management practices. Section 3 proposes and discusses the new methodological roadmap with 6 domains and 3 levels of intervention. Section 4 reports the data collected related to the guidelines and policies analysed and categorized according to the roadmap proposed in Section 3. Section 5 undertakes a cross-country analysis and discussion. Section 6 concludes the paper.

## 2. Literature review

### 2.1 Workforce is ageing

The worldwide worker population age is growing rapidly, and institutions, governments, and companies are being tasked to find new approaches to manage worker age-related trends.

**Figure 1.**
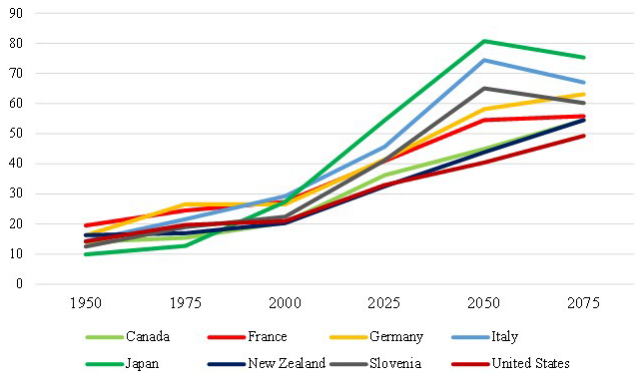
Percentage of demographic old-age dependency ratios: historical and projected values (1950–2075). Source: United Nations 2022.

Figure 1 indicates how aging population trend will continue in the next years in the studied countries and help us to compare it with historical values. Here, the old-age dependency ratio refers to the number of individuals aged 65 and older per 100 people of working age, defined as those aged between 20 and 64. The evolution of old-age to working-age ratios depends on mortality rates, fertility rates and migration. This indicator is measured as a percentage. Looking at Figure 1, it is predicted that the old-age dependency ratios would increase significantly in the 8 countries investigated in this work, and particularly so for Japan, Italy, and Slovenia.

**Figure 2.**
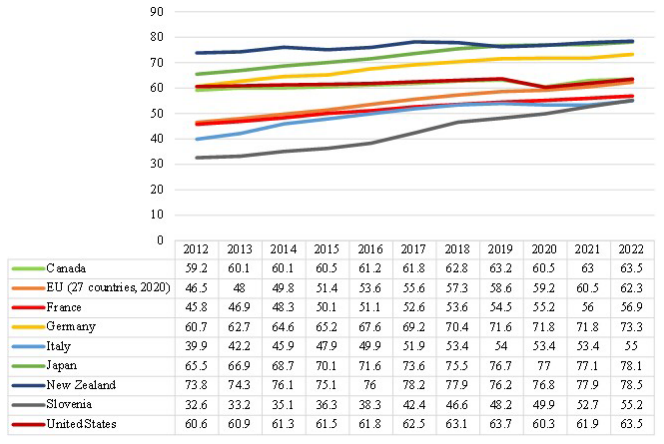
Employment rate by age group (in %), 55–64-year-old (2012–2022). Source: OECD, 2022.

To better understand the situation in the 8 studied countries, Figure 2 provides useful information about the percentage of the employment rate for a given age group (55–64) during the last decade (2012–2022). The employment rate for a specific age category is calculated by dividing the total number of employed individuals in that age group by the total number of individuals in the same age group, expressed as a percentage. According to Figure 2, we have witnessed an upward trend in the rate of ageing employment from 2012 to 2022, which indicates the ageing workers involvement has increased over the last decade. The aging employment rate is very high in some countries such as New Zealand and Japan with an average of 76% and 73% respectively over the last ten years. The employment of aging workers has been fairly stable during the years in some countries, such as in the United States, while in other countries like Germany, Italy and Slovenia the trend of ageing employment has increased gradually over the last ten years. In any case, it can be concluded that a wide range of countries have faced the ageing workers problem over the last decade, particularly in recent years.

### 2.2 Ageing workforce management

Ageing workforce management is a broad topic that contains different classifications according to the types of workplaces and industrial sectors under consideration. Moreover, managing an aging workforce always requires addressing unique challenges. Previous studies have placed much attention on some specific aspects rather than on a full comprehensive approach. In this section, the most relevant aspects regarding aging workforce management are discussed based on the published literature and this analysis will support the full understanding of the roadmap developed by the authors and discussed in the next paragraph.

On one hand, after the Covid-19 pandemic, the Eurozone and US businesses are facing unprecedented labour shortage, and on the other hand, the economic pressures of the worldwide demographic trends are straining pension systems, leading to a situation where workers are increasingly inclined to delay their retirement ( Case *et al.*, 2015). Delaying the retirement age increases the risk of being exposed to hazardous working conditions and, as a result, the chance of getting occupational diseases in businesses that require large numbers of workers ( Varianou-Mikellidou *et al.*, 2019). As workers get older, a loss in their cognitive and functional capacities may make many of the tasks they performed efficiently harder to perform ( Choi, 2015). The decline in physical capabilities among ageing workers, particularly in physically demanding industries, coupled with occupational risks, often lead to significant challenges such as early retirement or workforce losses. Therefore, industries must develop specific strategies for older workers to allow them to remain as long as possible at the workplace, prioritizing their safety and well-being ( Caporale *et al.*, 2022). In literature, ageing is often linked to physical capacity and mental health decrement ( Mcphee *et al.*, 2016), a significant loss in muscle strength ( Goodpaster *et al.*, 2006; Thompson *et al.*, 2015), and in flexibility and mobility ( Choi, 2015). Age-related changes in the locomotor system, including muscles and joints, indeed elevate the risk of Musculoskeletal Disorders (MSDs) ( Claudon *et al.*, 2020), particularly in manual and high physical demand tasks seen in manufacturing systems and industrial environments. These physical and mental changes can significantly affect the balance between job requirements and individual job capacity, especially when the task is highly physically demanding ( McGonagle *et al.*, 2015).

Aging has psychological implications. Older workers may feel uncomfortable in not being able to do certain tasks, which can negatively affect the worker’s psychological aspect (mental and social health). The opportunity and ability to continue working confers a sense of being useful and self-worth benefiting both the company (in terms of fewer disabilities) and society (in terms of healthier ageing) ( Gruenewald *et al.*, 2007). Employers must adopt age-friendly policies and practices to meet and accommodate older workers needs in terms of physical and cognitive capabilities ( WHO, 2015). Leveraging the strengths and capabilities of both younger and older employees can create a dynamic and complementary work environment. Some previous research as Börsch-Supan & Weiss (2016) and Göbel & Zwick (2013) highlight the potential advantages of mixed-age teams, where the unique competencies of each group can contribute to overall performance. Encouraging extended workforce participation can alleviate the burden on pension systems and enhance overall economic stability ( Holzmann, 2005).

In this context, research and initiatives focused on aging workforce management play a pivotal role in shaping policies and practices that optimize the potential of older employees while ensuring their well-being and contribution to society. By fostering age-friendly workplaces, implementing tailored training programs, and designing effective retirement policies, organizations can create environments where both older and younger employees thrive. In this regard, numerous organizations, and institutions such as the “World Health Organization (WHO)” and the “International Labor Organization (ILO)” offer guidelines and recommendations to companies that promote the active aging of their workforce. For example, the “Occupational Health and Safety Management System (OHSMS)” proposed guidelines for the promotion of active ageing, as the potential benefits derived from the older adult population are closely tied to their overall well-being ( Who, 2016). According to the ILO (2019), it is recommended to invest in ergonomic enhancements for workplaces, as well as to create a more favourable working environment. This will facilitate the ongoing engagement of older workers in the labour market. According to the EU-OSHA (2016a), age is characterized as a complex process including various dimensions such as biological, psychological, and social changes. These changes align with a decrease in both physical and emotional well-being.

### 2.3 Workforce diversity

Workforce diversity presents ongoing challenges and benefits to organizations. Researchers are currently emphasizing workforce diversity as a means to a more inclusive worker profile ( Shore *et al.*, 2018). In 2011, the Office of Personnel Management provided a comprehensive definition of “diversity” as the “characteristics such as nationality, language, race, colour, age, gender, disability, ethnicity, religion, socioeconomic status, and family structure”. Diversity, as defined by Hays-Thomas and Bendick (2013) refers to the combination of characteristics present in a workforce that have a substantial impact on individuals' thoughts, emotions, and behaviours in the workplace, as well as their acceptance, performance, satisfaction, and advancement within the organization. This issue becomes even more relevant since diversity in the workforce has increased significantly and this diversity may lead to social exclusion and inequality in the workplace ( Mor Barak *et al.*, 2001). Exclusion and discrimination in workforce management can detrimentally impact the psychological and physical well-being of workers, regardless of whether it is overt or subtle ( Jones *et al.*, 2013). Therefore, combating age discrimination is one of the preliminary steps for each organization to manage the aging workforce.

The professional and practical experience, specific knowledge, and skills possessed by aging workers make them a valuable asset for national and global economic growth ( Bogataj *et al.*, 2019; Dimian *et al.*, 2016). They possess a deep understanding of their work environment, know how to execute complicated tasks, and acquire mastery in manual industrial settings. These attributes along with an improved safety mindset and a heightened awareness of using personal protective equipment, make older workers more readily recognize potentially hazardous circumstances. Within several industries, older workers are highly valued by organization due to their loyalty, experience, and autonomy ( Case *et al.*, 2015; Choi, 2015; Kralikova & Koblasa, 2018; Kurtzer *et al.*, 2020). The study conducted by Calzavara *et al.* (2020) highlights that experience plays a crucial role in shaping the worker’s performance. Aged workers can compensate physical decline by leveraging their expertise to pass on their knowledge and skills to younger colleagues ( Eaves *et al.*, 2015). Using ageing workers' expertise as experience can have a positive two-sided effect on both their physical and psychological aspects since they are less involved in physical tasks but at the same time, they feel good to be used as trainers for less experienced workers ( Katiraee *et al.*, 2021b; Katiraee *et al.*, 2021c; Katiraee *et al.*, 2023).

 However, older workers, despite their greater expertise compared to younger workers, may encounter difficulties while doing physically demanding tasks that require high levels of pressure, involve repetitive actions, and have short time cycles, such as certain assembly tasks. Peruzzini and Pellicciari (2017) highlighted the importance of adaptive manufacturing systems. These systems are designed to accommodate the needs of aging workers, taking into account their declining physical and cognitive abilities. The ultimate goal is to enhance the interaction between humans and machines and improve the well-being of the workers.

According to the previous studies there are two different theories regarding ageing workers. One argues that an ageing worker positively affect human performance due to their experience and operational wisdom, while the other highlights the negative effects of aging on functional capacities, as physical and cognitive resources decline. Research has advanced a balanced perspective. For example, Boenzi *et al.* (2015) investigated the relation between functional decline and the experience of older workers using compensation theory. This theory suggests that ageing workers rely on their experience to compensate reduction in physical capacities. They developed an age-related model that considered age as a factor to determine the optimal job rotation schedules in work environments characterized by low load manual tasks with a high frequency of repetition (e.g. assembly lines). Similarly, Battini *et al.* (2022a) proposed a multi-objective job rotation approach to determine the optimal allocation of tasks and individualized rest-break plan for workers. This approach considers different socio-technical factors linked with age including workers' experience, physical capacity and limitations, postural ergonomic risks, and levels of boredom. Another study by Quintana, Leung, and Chen (2008), measured the effect of worker age and years of experience on the output and efficiency of manual electronics assembly. The findings showed an interaction between age and years of experience and their asymmetric impacts on assembly yield and time in the manual electronics assembly process. Therefore, while experience and a greater capacity to learn can enhance efficiency in many cases, the impact of aging on physical and cognitive abilities can complicate the relationship between experience, learning, and assembly time. It's essential for employers to provide appropriate support and accommodations to older workers to help them maintain productivity and job satisfaction as they age.

Most recently, based on a general awareness of increasingly closer human-machine interactions, Berti *et al.* (2023) presented a human digital twin architecture to improve worker-machine and worker-robot cooperation in shared and collaborative environments. According to the human digital twin concept, workers performance, health status, cognitive and ergonomics data and evolution could be available in real time for supporting analysis, simulation, and task scheduling, especially for ageing workers ( Berti *et al.*, 2021a and Berti *et al.*, 2021b). The worker-robot collaboration can ease the worker’s physical demand and provide an ergonomic environment which can be useful for older workers in manufacturing systems ( Keshvarparast *et al.*, 2021; Keshvarparast *et al.*, 2023a; Keshvarparast *et al.*, 2023b).

Such studies highlight the need for considering the specific challenges faced by aging workers in manufacturing systems and developing strategies to optimize the performance and well-being of a diverse workforce. Adaptive manufacturing systems, individualized and diversified job plans, job rotation schedules, and other interventions may be necessary to address the physical and cognitive decline associated with aging while leveraging the experience and expertise of these workers.

## 3. Roadmap for implementing an age-inclusive workforce management practice

This study proposes a new methodological roadmap with 6 action domains and 3 different levels of intervention in order to guide and support executives in implementing age-friendly workforce management in their companies.

Research on the management of an aging industrial workforce still appears to be in a pre-paradigmatic state ( Thomas, 1962), being various and diverse in terms of methods, concepts, and areas of focus. For example, Walker (2005) analysed age management dimensions at the organizational level to provide workers with opportunities for a flexible workplace, promotion of older workers and ergonomic workplace design. Streb *et al.* (2008) introduced organizational action fields to effectively handle the challenges given by an aging workforce. These action fields include “managerial mindsets”, “knowledge management”, “health management”, “human resource management”, “work environments and physical tools”. More recently, Deller *et al.* (2018) presented a tool named “Silver Work Index (SWI)”, designed to help organizations evaluate their capabilities in effectively employing older workers. SWI index comprises elements of organizational culture, leadership, and specific human resource (HR) practices, which are conceptually defined, but are yet to be operationalized for usage as an assessment tool. Following, Wilckens *et al.* (2020) presented an effective index called the “Later Life Work Index (LLWI)” to evaluate the readiness of companies for the ageing workforce emerging trend. This index considers 9 different dimensions with a focus on continued employment methods (independently of a particular retirement age). However, attempts to validate the two indices revealed country-specific variances due to differences in laws and regulations that complicated definition, comparison, and standardization.

**Figure 3.**
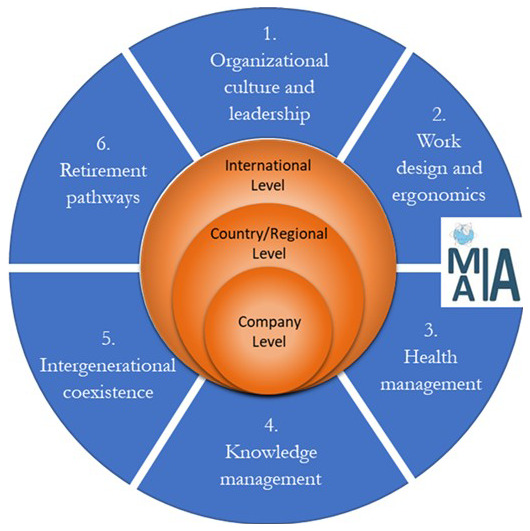
Roadmap for implementing an age-inclusive workforce management practice in worldwide companies.

We propose ( Figure 3) a new and comprehensive easy-to-use roadmap with a more parsimonious 6 main action domains and 3 levels of interventions. The roadmap draws on the afore-reviewed papers together with the recent “ISO 25550:2022” and the international guidelines developed by the “World Health Organization (WHO)”, the “International Labor Organization (ILO)”, the “European Agency for Safety and Health at Work (EU-OSHA)” and the “Occupational Health and Safety Management System (OHSMS)".

Each domain proposes an important managerial action to overcome known barriers and issues related to ageing workers in workplaces. All of them are necessary to consider in order to achieve a fully comprehensive and effective age-oriented management of the workforce. The degree of emphasis depends on various considerations, and in particular, the type of work environment. For example, in manual workplaces, the ergonomic aspect can be more important in manual work, while safety could be more important while working with devices.

Domain 1 “organizational culture and leadership” is typically the first to be implemented by managers by following a top-down approach, before starting the implementation of the other 5 domains depicted in Figure 3. The LLWI structure ( Wilckens *et al.*, 2020) too suggests that managers must take the lead in promoting an age-inclusive leadership and organizational culture in their companies. Considering the first domain, which helps companies’ culture improvement, a company can find its appropriate implementation sequence of the other 5 domains, according to its specific industrial sector needs, workforce characteristics and environmental factors. The 6 action domains also have several interrelations and mutual effects amongst themselves. Investing in one domain could mean positively affecting another one, and for this reason, a company should carefully select which investments and activities perform in order to achieve the best results on the majority of the 6 domains in the roadmap.

The domains here are universalistic in concept but we recognize that their prioritization would depend on the specific needs, goals, and type of companies. For example, the first domain (Organizational culture and leadership) is the foundation for accommodating ageing workers and fostering diversity in organizations. This domain requires time and effort, rather than a significant financial investment. The domains 2 (Work design and ergonomics) and 3 (Health management) need more investment in terms of both cost and time. They are more complex since workers may need different requirements according to their differences. Furthermore, domains 4 (Knowledge management) and 5 (Intergenerational coexistence) are more difficult and tactical to execute in terms of speed of implementation, while retirement pathways (domain 6) are relatively simple to develop. Hence, it is crucial to consider that while some domains may be more challenging, they are equally important in supporting aging workers and promoting a diverse and inclusive workplace.

The six domains proposed in this framework, together with a brief listing of keywords and research topic, are outlined below:

### 3.1 Organizational culture and leadership

Since age can be considered as among the most important dimensions of worker diversity, it is crucial for organizations to manage this diversity well. Initiatives range from improving organizational culture and leadership to support equality of opportunity ( Deller *et al.*, 2018), reducing discrimination ( Button, 2020), creating a positive image of the aging workforce ( Wilckens *et al.*, 2020), rewarding older workers’ achievements, and actively considering workers’ preferences, viewpoints and wishes ( Carstensen *et al.*, 1999). Literature suggests that an age supportive organizational culture and climate not only diminishes potentially negative views about specific age groups but also reduce tensions and conflict among different age groups ( Burke, 2015). According to various studies ( Armstrong-Stassen, 2008; Armstrong-Stassen & Schlosser, 2011; Cheung & Wu, 2013; Hennekam & Herrbach, 2013), the combination of organizational culture and leadership were considered the primary factor for achieving successful and motivating workplace, both leading up to and continuing beyond the retirement age. This is further supported by additional factors that collectively contribute to a supportive workplace environment including work design, health management, individual development, knowledge management, transition to retirement phase, and employment during retirement phase. This aspect becomes even more urgent for company managers, since they are experiencing an increase in labour shortage, especially for younger workers.

### 3.2 Work design and ergonomics

**Figure 4.**
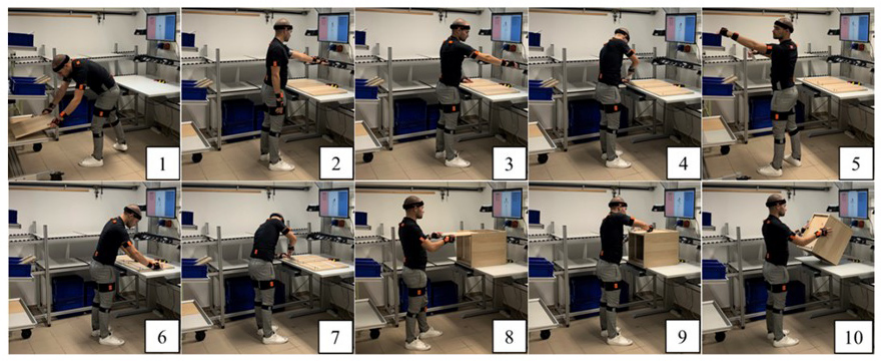
Ergonomic assessment of ten different postures using the digital ergonomic platform WEM (This figure/table has been reproduced with permission from Battini *et al.*, 2022b).

Ergonomic solutions and human-centric workplace designs are increasingly used by organisations to promote efficiency and maintain the employability of the workforce, especially in labour intensive manufacturing settings ( Thun *et al.*, 2007). A study conducted in Germany found that older workers have a greater relative contribution to productivity when companies offer specific equipment, establish employment positions tailored to the age group, and implement mixed age working teams ( Heywood & Jirjahn, 2016). According to the European Commission guidelines, countries should apply reliable ergonomics principles more effectively to the way in which workplaces are designed and work is organised in order to avoid accidents, occupational illnesses and workforce demotivation. Improving the work environment for ageing workers is possible in several ways, especially in manufacturing and logistics sectors. Operations managers should re-think the workspace according to human centric paradigms ( Battini *et al.*, 2018 and Berti *et al.*, 2022), carefully considering the ergonomics of the manufacturing process in terms of postural assessment and fatigue level measurement ( Figure 4), well understand the role and impact of Industry 4.0 technologies on ageing workers ( Calzavara *et al.*, 2020 and Neumann *et al.*, 2021) and, finally, invest efforts towards the creation of flexible work plans and individual-oriented job scheduling programs ( Battini *et al.*, 2015; Battini *et al.*, 2016; Battini *et al.*, 2022a; Berti *et al.*, 2023; Katiraee *et al.*, 2023).

### 3.3 Health management

Health management is an important element of contemporary work environment. Employers are legally required to take all necessary measures to promote employee health. Occupational safety and health (OSH) laws are usually aimed to prevent hazards and to build an organizational health culture that prevent accidents at work and harm to employees from their work activities. The working life should be self-adapted to the capabilities and current situation of the worker (mindset, wellbeing, concentration) in order to create a working environment, which provides an optimum of high productivity together with best health conditions at the same time ( Deller *et al.*, 2018; Oliveira, 2018). Actions dedicated to this domain would implement projects and investments that aim to improve workers’ physical health and well-being, by a working environment analysis and assessment, illness and injury rates analysis in relation to the profile of age-related patterns, and systemic addressing of health risks, physical and psychological (mental and social health) improvements ( Wilckens *et al.*, 2020). According to research by Olesen *et al.* (2012), early retirement is linked to both poor physical and mental health.

Domains 2 and 3 (work design and health management respectively) are mutually and naturally linked and investing in one of them implies improving the other by positively affecting the well-being of the ageing workforce. However, while domain 2 is mainly focused on workplace design for ergonomics and human centric aims, domain 3 is concerned with wider actions related to all aspects of work life that affects human health, well-being, and behaviour.

### 3.4 Knowledge management

Within the action domain 4, particular emphasis is devoted by companies on the succession planning process, mainly related to hiring and retaining employees and on the overall knowledge management process, namely knowledge creation, knowledge storage, knowledge transfer and knowledge implementation between different worker generations, already retired workers ( Deller *et al.*, 2018). Indeed, Deller *et al.* (2018) proposed that knowledge management is a fundamental component of the index for active aging in organizations. Information-communication technology acts as an enabler to facilitate especially knowledge storage and knowledge transfer. Actions implementable in this domain are as follows: succession planning process re-thinking and re-planning (related to hiring and retaining), knowledge storage, transfer and exchange, conservation of accumulated knowledge between different generations (from young to older and from older to younger). In this last context, the adoption of specific last generation technology (as AR and VR for examples) to help knowledge exchange and storage and expertise transfer is gaining momentum ( Ellwart *et al.*, 2013). This domain concerns creating a working environment that supports learning, knowledge sharing, worker development and mentorship. Domain 4 may thus have a direct impact on domain 5 (Intergenerational coexistence) and are mutually reinforcing. The significance of work design and health management increases as individuals age, whereas dimensions such as knowledge management and individual development should be customized to suit older employees ( Deller *et al.*, 2018).

### 3.5 Intergenerational coexistence

This action domain seeks to build a ground up culture of active engagement among worker generations ( Barabaschi, 2015; Méda *et al.*, 2017). In contrast with top-down actioned domains, the intergenerational coexistence domain is oriented towards bottom-up implementation. It provides enabling mechanisms such as multigenerational working teams, incentives, and channels for exchange of ideas and communication amongst workers of different ages, and training programs and development solutions involving young and old workers (coaching and mentoring sessions).

Currently, four or sometimes five generations are present in the labour market, working together (Traditionalists, Baby Boomers, Generation X, Millennials and Generation Z). The multigenerational workforce encompasses a more diverse range of workers across all age groups compared to the past when workplaces were predominantly composed of larger numbers of younger workers and fewer older workers. The presence of a multigenerational workforce offers companies an opportunity to leverage the diverse range of experiences and talents that different age groups possess, hence enhancing productivity and profitability. By implementing appropriate workplace practices and utilizing effective tools, organizations can enhance their agility, inclusivity, and overall effectiveness. The primary benefit of a multigenerational workforce is the facilitation of effective synergy between experienced and less experienced staff, resulting in advantages for both employers and employees ( Katiraee *et al.*, 2021c; Mejaš *et al.*, 2014). Costanza & Finkelstein (2015) argue that designing policies based on certain generations may not be the best approach. Instead, workforce individualization in terms of age identifies the needs of each worker individually and it is the useful approach to manage employees ( Smeaton & Parry, 2018).

### 3.6 Retirement pathways

One of the main issues to address with an aging workforce is retirement and succession pathways ( Crumpacker & Crumpacker, 2007). As older employees approach retirement age, organizations must plan for their eventual departure and ensure a smooth transition of knowledge and expertise to younger employees. Job departure times vary. For example, Denmark is anticipated to have the highest retirement age in Europe, with both men and women expected to retire at the age of 74. In the European Union, the majority of Member States have set the legal retirement age at around 65, fluctuating from 62 to 67 ( www.oecd.org). The thrust seems to be towards postponing retirement (and reduce pension and associated state or corporate burdens). For example, Emmanuel Macron recently proposed his somewhat controversial plan to raise the official retirement age in France from 62 to 64. The USA does not insist on a legal retirement age, barring selected professions such as army, police or firefighters. Social security in the US activates at different ages depending on one’s date of birth – 62 years is the minimum age for eligibility currently, albeit with reduced benefits. New Zealand does not prescribe an official retirement age, even if the common age to retire is 65 when NZ Super and some other pension payments start ( www.govt.nz). In Canada, the standard age to start the pension is 65 years. However, the maximum monthly amount of pension is reached when one turns 70 ( www.canada.ca). In Japan, due to the increase in the public pension age to 65, those who retire at age 60 would experience a period of zero income. Currently, a re-employment method is being utilized to address the gap. According to the data, the average effective retirement age for Japanese males is 69.5 years, while for females is 66.5 years. These are the oldest ages for regular, recorded employment among developed countries ( www.oecd.org). Succession planning in anticipation of retirement of critical expertise becomes crucial in companies to avoid knowledge gaps and maintain organizational continuity. Companies’ actions required in this domain are primarily devoted to retaining workers at work as much as possible according to their capabilities and wills: flexible work arrangement, personal life supports, re-skilling and abilities improvements ( Halpern, 2005; Ulrich & Brott, 2005). In addition, actions devoted to build senior workers’ entrepreneurship, self-employment, career development, and other transition solutions to retirement are useful in this domain (i.e. Bogataj *et al.*, 2019).

The roadmap ( Figure 3) additionally introduces three-levels of interventions in regard to the 6 actions domains:

- *International Level*: This level includes international standards developed by institutions (EU, ISO etc.) to guide and standardize international policy and practice in ageing workforce management.
- *Country/Regional Level*: This level includes laws, policies and guidelines developed in a particular area (e.g., internally developed in a country, or a region or a province of the territory) to help managers in developing and implementing ageing workforce management policy and practice.
- *Company Level*: This level includes strategies, guidelines, best practices, or case studies developed in specific companies with the aim of retaining aging workers by helping maintain their work ability, safety, and self-respect.

## 4. Dataset collection of age-related policies and best practices

The roadmap proposed in the previous paragraph provided conceptual guidance for data collection, and a categorization and comparison analysis among the 8 countries involved in the international European Project MAIA (H2020-MSCA-RISE, www.maiaproject.eu). The 8 countries involved in the project and investigated by the authors are: Canada, France, Germany, Italy, Japan, New Zealand, Slovenia, and USA.

The roadmap provides a structure to examine the best-practices and policies analysed by the authors during the project activities according to the 6 action domains and 3 levels of intervention depicted in the roadmap ( Figure 3). The full dataset collected by the authors is reported in our repository (Tables A1, A2, A3 in Katiraee *et al.*, 2024). In the following paragraphs, the data collected and reported in repository will be discussed according to the three levels of the roadmap: international level, country/regional level, company level.

### 4.1 International level

There are ageing workforce-oriented policies that have been defined at an international level, for example, for all European countries or for all countries in the world. The European level might involve the European Union (EU) and its agencies working on employment and social affairs. They may have specific directives and policies addressing the aging workforce for all EU member countries. Similarly, other regions like the Americas or the Pacific may have their own regional organizations or agreements that address aging workforce management with a focus on the unique needs of those regions.

In this section, we summarize the existing policies at the international level for managing ageing workforce (more details in Table A1 available in Katiraee *et al.*, 2024).

Starting from EU strategies and policies, “ *The EU strategies on OSH and the ageing labour force / The project ‘Safer and Healthier Work at Any Age*” was developed in 2002 for the first time. “ *Community Strategy on Health and Safety at Work (2002–2006)”* represented a change towards a comprehensive approach as stated by the European Commission. It acknowledged the aging of the workforce and emphasized the necessity of addressing the issue of demographic transition. The strategy implemented a comprehensive approach to workplace well-being, considering the evolving nature of work and the rise of new psycho-social dangers. In addition, they focused on the physical, moral, and social aspects of work instead of just emphasizing accident prevention and occupational health. This EU strategy has evolved and expanded over a time with different initiatives as below:

1. *“The 2007–2012 OSH strategy”:* This strategy included the rising prevalence of musculoskeletal disorders (MSDs) and the excessive exposure of susceptible populations, such as older employees, to industrial hazards. To address this issue policies on safety and health at the workplace were developed which are more effective for individual needs; in addition, the strategy aimed for a 25 % reduction in the overall occurrence rate of workplace accidents by 2012 across the European Union's 27 member states, through the enhancement of health and safety measures for workers. The strategy also aimed to enhance the use of reliable ergonomics principles for designing works and workplaces. in which workplaces are designed and work is organized.
2. *“The 2013 evaluation of the European 2007–2012 strategy”:* The 2013 assessment of the European 2007–2012 strategy yielded significant findings and suggestions for the new upcoming EU safety and health strategy. One of the five primary concerns in the field of safety and health at work is the ageing and decline of the European workforce. It emphasizes the importance of implementing measures to keep older workers employed. The objective of the strategy was to achieve a 25% decrease in the overall occurrence rate of workplace accidents by 2012 in the European Union, namely in the 27 member states, by the enhancement of health and safety measures for workers.
3. *“Strategic Framework on Health and Safety at Work 2014–2020”:* The EU’s Strategic Framework on Health and Safety at Work 2014–2020, released in June 2014, seeks to pinpoint significant challenges and strategic goals for health and safety in the EU workplaces. It outlines essential measures to safeguard workers' health and safety and identifies the tools necessary to accomplish the framework's objectives. The approach sought to enhance the enforcement of current health and safety regulations and address novel and emerging hazards to avoid occupational illnesses, while also addressing ongoing concerns.
4. *“The Strategic Framework on health and safety at work 2021–2027”:* This strategy outlines the European Commission’s vision to enhance workers’ safety and health, as well as to meet one of the pledges of the European Union to update worker protection regulations and address both traditional and emerging work-related risks.
5. *“Employment initiatives for an ageing workforce”:* There has been a significant increase in policy focus and consideration of the aging workforce during the past decade. At the European Union level, there are concerns regarding the long-term viability of pensions, economic growth, and the availability of labour in the future. These issues have led to various policy procedures and suggestions aimed at promoting longer working lives and delaying retirement. Over the next two decades, there will be a shift in the age distribution of the working population, resulting in a decrease in the overall number of individuals of working age. There are numerous challenges:
  ⚬ to maintain and enhance the physical and mental well-being of workers as they get older.
  ⚬ to enhance the expertise and employability of older workers.
  ⚬ to create appropriate working environment and job prospects for an older worker.
  This action plan aims to enhance employment of older workers through the implementation of government policies, engagement of social partners and social dialogue, and active involvement of companies and older workers.
6. *“European Parliament pilot project on health and safety of older workers / The three-year pilot project, Safer and Healthier Work at Any Age (2012)”* is another project for ageing workers. The European Parliament launched a three-year pilot project, overseen by EU-OSHA, to address occupational safety and health (OSH) concerns of older workers. This project focused on issues such as rehabilitation and return to work. The project aims to evaluate the necessary conditions for OSH strategies and systems in different EU Member States to address the challenges posed by an older worker and to ensure better prevention for all throughout working life.
7. *“Guiding Principles for Active Ageing and solidarity between generations, Council Declaration on the European Year for Active Ageing and Solidarity between Generations (2012): The Way Forward”.* The purpose of these principles and guidelines is to promote equal rights for older workers in the labour market, eliminate the use of age as determining factor in assessing a worker’s suitability for a job, prevent negative age-related stereotypes and discriminatory attitudes towards older workers at the workplace, and recognize the valuable contribution made by older workers. Furthermore, these principles prioritize the advancement of working conditions to accommodate the evolving requirements of older workers, thus preventing early retirement. And finally, they provide opportunities for education, training, and skills enhancement, enabling older workers to re-enter and fully engage in the labour market.
8. *“PES Strategies in Support of an Ageing Workforce”:* The EU developed PES (Public Employment Services) strategies in 2019 to make improvement in the human relations practices. These adjustments are intended to enhance the employability of workers who are currently employed.

Other policies developed at an international level are as follows:

9. *“ISO Technical Committee 314 on Aging Societies and the new-born ISO 25550/2022”*. The ISO worldwide standards offer guidance for companies and stakeholders to create, execute, sustain, and endorse a **n** age-inclusive workforce. This initiative aims benefit the organization, older employees, communities, and other stakeholders. Organizations have the option to use this guideline either independently or as or as part of their management systems, human resources initiatives, corporate social responsibility, or diversity and inclusion programs.
10. *“DEI framework for Diversity, Equity and Inclusion”.* Born in US during the 1960’s, it now comprises all the international initiatives focusing on policies, practices, and culture to correct inequities within an organization or a business. The focus on ageing society and ageing workforce is still very weak in the DEI movement.
11. *“ESG investment evaluation criteria”:* Environmental, social and governance (ESG) is a set of criteria used to evaluate a company’s performance in terms of its governance mechanisms and its ability to successfully manage its environmental and social impacts. Also, in this case, as for the DEI framework, the focus on ageing workers specific needs and characteristics is still very weak.

### 4.2 Country level

In this paragraph, we concentrate on the age-friendly policies developed by the 8 countries involved in this analysis: Canada, France, Germany, Italy, Japan, New Zealand, Slovenia, and United States of America. The analysis aims to highlight which countries have already started to develop age-friendly policies and guidelines along the six domains presented in road map in Figure 3.

In Table 1, we report the 74 age-oriented policies we were able to find concerning country level initiatives per each country. Table 1 does not represent the total number of age-friendly standards, laws, policies, and guidelines that exist in each country, it would rather report the number of contributions that we had easily access and we were able to analyse for this benchmark analysis. More details regarding the country level policies can be seen in Table A2 available in our repository ( Katiraee *et al.*, 2024).

**Table 1.** 74 age-oriented policies analysed per each country involved in the MAIA project.

Country	# of occurrence
Canada	15
France	9
Germany	13
Italy	4
Japan	8
New Zealand	12
Slovenia	5
USA	8

**Figure 5.**
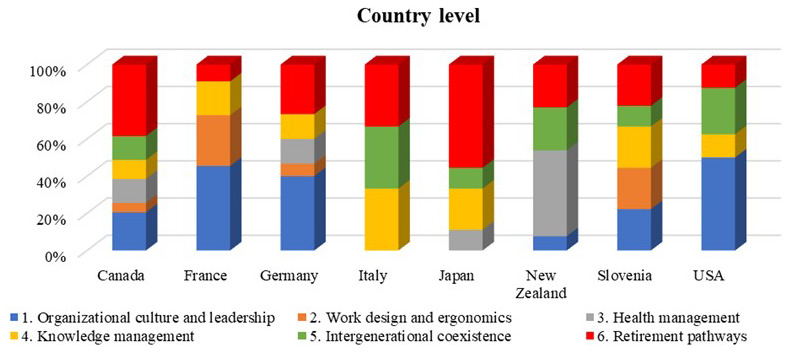
Age-oriented policies per country and roadmap domain.

The following bar chart ( Figure 5) represents the distribution of the age-oriented policies according to the six different domains of the roadmap per each country. The distribution that characterizes the countries provides information regarding the commitment that the country is putting on a particular topic toward the design of ageing workforce management practices.

The results of this analysis showed that the first domain of the roadmap, named “Organizational culture and leadership”, was addressed by almost all countries by developing specific age-oriented policies, except in Italy and Japan.

Furthermore, we could notice that the domain related to “Work design and ergonomics” (domain 2) was rarer, since age-oriented policies for ergonomic design of the workplace are usually derived from international standards (ISO 11228) methods for ergonomics design, that are conceived for any kind of workers. Only in France and Slovenia we found specific age-oriented workplace design guidelines.

The domain related to “Health management” for ageing workers (domain 3) was seen to be is quite developed for New Zealand and Japan, while “Knowledge management” (domain 4) is evenly attended among the partners: in fact, we were able to find some best practices and flexible retirement policies that facilitate the transfer of knowledge between ageing workers and new employees.

The domain related to “Intergenerational coexistence” (domain 5) represents an area that has partially been investigated for the analysed countries, with different country level initiatives, in particular in Italy and US.

Finally, “Retirement pathways” (domain 6) was the only domain where we did not face difficulty in obtaining information, since it appears to be a common programmatic initiative to foster ageing workforce retention in manufacturing work field.

The following sections provide some general insights concerning age-oriented initiatives in each country.

***4.2.1 Canada.*** Canada is comprised of ten provinces and three territories, each functioning as sub-national administrative divisions under the Canadian Constitution's authority. These provinces each have unique histories, particularly concerning policies on ageing, notably in the realm of mandatory retirement regulations. The Canadian federal government abolished mandatory retirement for its employees in 1986. In a significant move, Ontario's government enacted Bill 211 in 2005, known as the Ending Mandatory Retirement Statute Law Amendment Act, 2005, thereby making age-based discrimination illegal under the Code. Similarly, the Alberta Human Rights Act prohibits employment discrimination based on age, effectively rendering mandatory retirement at 65 as a form of age-based employment refusal. There's notable variability among provinces concerning enforced retirement rules. For instance, Manitoba and Quebec abolished these rules in the 1980s, whereas Alberta, Newfoundland and Labrador, and Nova Scotia made amendments much later, between 2000 and 2009 ( Messacar & Kocourek, 2019). During this transition phase in retirement age, Canada, alongside international bodies like the OECD, has been considering life-course approaches to policies that support older workers' participation in the labour force. Canadian provinces have been particularly attentive to transitions in the ageing workforce, focusing on aspects of work, education, family, and health ( Bélanger *et al.*, 2016). Strategies for easing into retirement include allowing employees to cut their workweek by up to 40% without impacting their benefits or pension. This approach facilitates knowledge transfer and quality family time ( Joyce, 2007). Although Canadian companies' webpages often focus on retirement policies, less attention is given to other significant aspects such as intergenerational relations, and the psychological and physical well-being of ageing workers ( Lagacé *et al.*, 2015). Notably, the Federal Employment Equity Act of 1995 outlines employment opportunities and benefits for various disadvantaged groups, including people with disabilities, women, aboriginal peoples, and visible minorities. However, contemporary definitions of disability do not encompass age ( McMullin & Shuey, 2006).

***4.2.2 France.*** The current debate in France centres around the retirement age, especially after Prime Minister Emmanuel Macron's recent proposal to increase it from 62 to 64. France's retirement system includes a state pension and a workplace-related pension plan. A 2010 reform suggested a gradual rise in the minimum age for pension eligibility from 60 to 62 and for receiving a full pension from 65 to 67. The state pension allows for early retirement at age 60 for those who have completed the required contribution period and began employment before turning 20. Similarly, the workplace pension plan permits early retirement, typically with certain deductions based on retirement age and contribution years. Transition to retirement focuses on transferring knowledge, and studies indicate that managerial and professional roles receive more training opportunities than manual labourers. For instance, a vast majority of senior managers and professionals, about 89%, were informed about the "Bilan de compétences," a skills assessment provided by the government in 2016. In contrast, only 38% of older manual labourers were given this information. This disparity results in manual labourers facing a decrease in employability sooner, with reduced training opportunities beginning at 35, as opposed to 50 for managerial and professional employees. Employability loss is also linked to job type and the health and physical capabilities of workers. The French Ministry of Labour (Ministère du Travail) plays a key role in occupational health and safety policymaking and works in collaboration with various social partners within the Steering Committee on Working Conditions to shape national health policy strategies (EU-OSHA).

***4.2.3 Germany.*** Investments in Germany's ageing workforce have led to significant shifts in part-time work and retirement trends, beginning with the endorsement of partial retirement incentives in 1996. These changes have resulted in an uptick in part-time work among older employees, particularly extending the working lives of women more so than men ( Berg *et al.*, 2020). The 2006 reforms, which shortened the duration of unemployment benefits, correlated with an uptick in employment among the elderly, especially in terms of the link between reduced unemployment benefit durations for older workers and both unemployment duration and labour market involvement ( Riphahn & Schrader, 2020). Furthermore, the German Federal Ministry of Labour and Social Affairs has introduced the Federal Program, established in October 2005 across all federal states, to enhance the employability of older individuals. Regarding retirement, the standard old-age pension in Germany starts at age 65, given at least five years of contributions. Many seniors opt to continue working due to longer life expectancy, healthier lifestyles, and sustained work capacity. Early retirement is available from age 63 for those with a minimum of 35 years of insurance contributions ( OECD, 2020). In terms of safety and health for older workers, German associations are dedicated to creating effective work environments through interdisciplinary research and design. The "Gesellschaft für Arbeitswissenschaft" (GfA) advocates for Human Factors and Ergonomics in German-speaking regions and on a broader European and international scale, offering platforms such as workshops, publications, and consultations to disseminate ergonomic knowledge. It has been shown that better education and training lead to more favourable conditions for retaining aging workers in their jobs, with higher qualifications and education being crucial factors for prolonged employment.

***4.2.4 Italy.*** In 2012, Italy and other partners in the MAIA project implemented regulations that provided more flexibility in the criteria for retirement age. These policies included incentives for prolonging one's career and penalties for early retirement. The qualifying age for retirement was raised but also made more adaptable. Specifically, the age range for men was set between 66 and 70 years, while initially it was 62 years for women, which was then progressively increased to 66 years by 2018 (OECD). Additionally, the Italian government launched initiatives in 2004 and 2007. The latter year saw the Italian Ministry of Labour, Health and Social Policy introduce a scheme focused on helping disadvantaged workers, particularly those aged 50 and above, to find employment again. This program, known as Programma PARI – azioni per il rimpiego (2007), offered special employment benefits and encouraged participants to engage in training, re-qualification, and job hunting. Furthermore, following the European Union's declaration of September 14, 2011, as the "European Year of Active Aging and Solidarity between Generations," Italy participated with its own initiatives. These efforts aimed at fostering a more positive perception of old age, emphasizing the importance of learning and training, facilitating knowledge transfer between the old and young, and addressing any existing or potential intergenerational conflicts.

***4.2.5 Japan.*** Our research reveals that Japan is focused on the retirement pathways domain. Late retirement is possible between aged 65 and 70. Since 2004, individuals over 65 can simultaneously earn a salary and receive a pension, provided their combined earnings and pension income do not surpass JPY 470,000. If this threshold is exceeded, the full pension is still paid, but half of the amount above the limit is deducted from the pension related to earnings. Additionally, workers who are over 70 years old are exempt from making contributions. This policy has resulted in Japan having the highest rate of employment among older adults within the OECD countries. In 2020, 50% of those aged 65 to 69 were employed. Additionally, the Japanese government offers different services encompassing consulting, guidance, and job placement for 55+ and 65+ years old, who are looking for job positions. To enhance the employment of ageing workers, the government also promotes flexible work practices and encourage employers to be productive in the design and development of best practices. In 1987, the Japanese government amendment the “Labour Standard Law” (1947), by which it requested organizations to design flexible working hours, including flex time and compressed work hours, although the adoption for employers was predominantly voluntary ( Flynn *et al.*, 2014).

***4.2.6 New Zealand.*** New Zealand does not have a mandatory or defined retirement age. The analysis performed for New Zealand highlighted a strong focus on the country on health management domain. Demonstrating its strong commitment, the Ministry and District Health Boards (DHBs) in New Zealand are actively engaged in disseminating information about aged care plans to the aging workforce and their families. This effort is aimed at enhancing awareness and helping them make informed decisions regarding self-care and available health and disability support services. Additionally, this plan encompasses addressing workforce development issues to cater to the increasing numbers of older Pacific peoples and members of other ethnic communities. New Zealand consistently shows a high level of concern for ethnic groups. For instance, the Ministry of Health and DHBs advocate for hospital-based healthcare services that are tailored to the needs of the elderly Māori population. A primary goal of New Zealand's public health service is to promote and safeguard health, with a particular focus on enhancing wellbeing in older age. This includes initiatives to improve nutrition, boost physical activity, and coordinate efforts across various agencies concerning housing and transportation. The New Zealand Government has also shown its dedication to managing an aging workforce through various campaigns. These include the creation and enhancement of employment services, the formulation of the New Zealand Income Insurance scheme, and the evaluation of active labour market programs (ALMPs). Furthermore, the government is encouraging employers to research and provide more opportunities for older workers, particularly in terms of flexible working arrangements. Special attention is given to ethnic groups, aligning with other employment action plans focusing on Māori, women, Pacific people, disabled individuals, recent migrants, and ethnic communities.

***4.2.7 Slovenia.*** Slovenia implemented a pension reform in 2020 to promote the participation of older workers in workplaces. This reform enables individuals to continue working beyond the age at which they qualify for an old age pension, without any restriction on their earnings or a limit on the amount which their pension payments are decreased. The 2020 reform brought some improvement in the flexibility to integrate employment and pensions as well as an increase the likeability of work even close to retirement age. While employed on a full-time basis, individuals are eligible to receive only 40% of their old-age pension for the first three years, and subsequently 20%. This indicates that there is a compulsory postponement of 60%, followed by an additional 80% reduction in the benefit when working. These regulations also apply to people who return to work after retiring ( OECD, 2022). The Active Aging Strategy designed by the IMAD - Institute of Macroeconomic Analysis and Development of the Republic of Slovenia promotes flexible working hours and appropriate working conditions. In particular, the strategy addresses older workforce needs by adapting jobs and processes due to technological progress and digitalization. Consequently, longer working lives for ageing workers is possible through continuous education and training and more flexible forms of work for older people and retirees.

***4.2.8 United States of America.*** The data collected United States of America (USA) age related policies shows an emphasis on organizational culture and leadership domain. In this regard, several laws were designed to avoid age discrimination inside companies, such as “The Age Discrimination in Employment Act (ADEA)” (1967) that bans age discrimination against employees who are 40 years or above. Moreover, to help business owners, managers, and government officials to in comprehending their legal responsibilities in preventing any kind of employment discrimination, Technical Assistance Programs (TAPS) were issued with a particular focus on age discrimination. With a special regard on people with physical and mental disabilities, the Americans with Disabilities Amendments Act (ADA) (2008) aims to protect affected people of all ages. USA also provides some initiatives special targeting ageing workers such as Senior Community Service Employment Program (SCSEP). In this last example, the Department of Labor program offers training, job placement aid and additional support services to unemployed aged 55 and above who have low income ( Collins & McCaskill, 2016).

### 4.3 Company level

The company level in our roadmap involves the programs, best cases and best practices related to the approaches and methods for aging workforce management developed by companies. In this regard, a cross analysis was performed among the 8 countries involved in this project: Canada, France, Germany, Italy, Japan, New Zealand, Slovenia, and United States of America. The analysis examines how companies in the above-mentioned countries address the ageing workforce problems and in which of the 6 defined domains. Table 2 below reports 95 company best cases gathered in our research. Table 2 is not exhaustive, reporting just the number of contributions that we could access and analyse. More details on the 92 company cases analysed can be seen in Table A3 in our repository ( Katiraee *et al.*, 2024).

**Table 2.** 92 age-oriented company best cases/practices analysed per country.

Country	# of occurrence
Canada	11
France	15
Germany	18
Italy	10
Japan	9
New Zealand	3
Slovenia	7
USA	19

The following charts represent the distribution of the information that we were able to collect on the six different domains per country.

**Figure 6.**
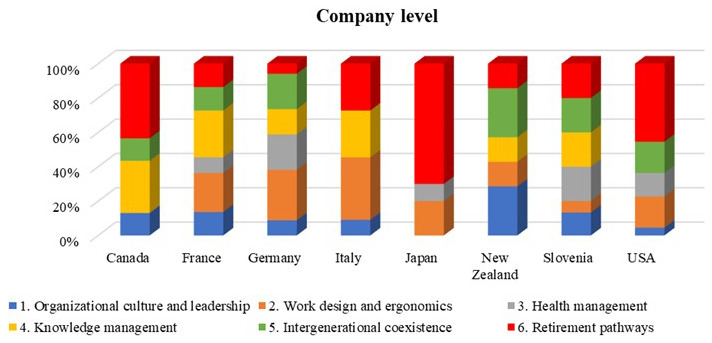
The distribution of ageing workforce management policies by companies in each country (Company level).

Figure 6 classifies the 92 best company cases according to domain and country. Our examination indicates an overall lack of priority for the organizational culture and leadership domain. For example, in Japan, none of the studied companies consider improving their culture towards ageing workforces.

A considerable proportion of company’s report investing in work design and ergonomics to manage their ageing workforces. This proportion is relatively high in Italy, France, and Germany, but not so in Canada. Work design and ergonomics primarily provide ergonomically designed workstations for all workers and rotate physically demanding or repetitive tasks. These simple but practical actions can have a positive impact on the health and safety of older workers.

Knowledge management is a priority item for companies in Canada contrast to the companies in the US and Japan. Other domains such as retirement pathways and inter-generational coexistence have been considered in all the studied countries.

Figure 6 suggests that companies in Germany have a good presence in all domains. A possible reason is that Germany experiences a significant decrease in its natural working-age population, and as a result there is a heightened focus on enhancing the integration of older workers into the labour market. Hence, workers in Germany continue to benefit from a substantial pay-as-you-go public pension program with elevated effective replacement rates and comparatively early retirement ages when compared to workers in many other nations. Furthermore, in most of the countries, especially in Germany, unemployed older workers continue to face severe difficulties in finding a new job. The challenges lead to elevated levels of prolonged joblessness among older employees in many European countries. In addition, about 52% of the studied companies in Germany, have considered more than one domain to manage their ageing workforce which shows that German companies pay attention to the problems of the ageing workforce in multiple ways. All domains are also considered by the studied companies in the France and Slovenia, which shows the concern of these companies about all defined ageing workforce management. The same trend can be observed in other studied companies in Italy, New Zealand, and USA, where most of the domains have been considered in managing ageing workers. Relatively fewer domains find frequent mention in companies in Canada and Japan. A lack of access to detailed company data could be a reason for this finding.

## 5. Discussion and international comparison

In the following paragraphs we discuss the data collected and analysed at the three levels of intervention described before with a focus on each action domain of the roadmap in Figure 3.

### 5.1 Domain 1: Organizational culture and leadership

Figure 5 indicates that a considerable proportion of the policies and programs at the country level are dedicated to organizational culture and leadership, particularly for countries such as Canada, France, Germany, Slovenia, and the USA. However, this emphasis declines significantly at the company level ( Figure 6). The data suggests that country level legislation and policies are not necessarily adopted at the company level, due to different reasons. For example, US companies may pay more attention to more tangible initiatives such as work design and ergonomics or retirement pathways rather than organizational culture, since the former may result in more immediate productivity gains and quantifiable avoidance of medical, legal, and reputational costs.

From a country level perspective, Europe has been comparatively late in addressing age discrimination. The EU adopted the Framework Directive on “Equal Treatment in Employment and Occupation, Council Directive 2000/78/EC” in Nov 2000 in contrast to the US which started from 1935 when provided old age assistance and old age survivors insurance. The EU Directive bars age discrimination in employment, vocational training, and social security. Employers cannot deny a job, promotion, or training on age related grounds. The EU has launched several initiatives to manage ageism, such as the “European Year of Active Ageing and Solidarity” between Generations in 2012, which aimed to promote intergenerational cooperation and combat stereotypes about aging. Efforts to manage age bias in the USA are seen in awareness campaigns, such as the AARP's "Disrupt Aging" initiative, which aims to challenge stereotypes about aging and promote the value of older workers in the workforce ( Jenkins, 2016). U.S. also provides assistance programs and resources, such as Technical Assistance Programs (TAPS) and Disability Employment Initiative (DEI). TAPS is specifically developed to provide human resource employees, business owners, managers, union officials, and government officials with an extensive understanding of their legal responsibilities in preventing all types of employment discrimination, including age-based discrimination. DEI concerns demonstration projects that improve existing programs for increasing employability for people with disabilities.

At the international level organizations such as the “International Labor Organization (ILO)” campaign for the protection of older workers and demands that employers should ensure that older workers have a fair chance of being employed and remaining competitive in the labour market. More recently (2019) the WHO launched global campaign to combat ageism, an initiative supported by WHO's 194 Member States. The WHO Campaign provides different resources (e.g., global report that provides overview of origins, types and measures) and toolkits aims to alter the prevailing discourse on age and the process of aging, with the ultimate goal of fostering an inclusive society that caters to individuals of all age groups.

### 5.2 Domain 2: Work design and ergonomics

Figure 5 and Figure 6 show a gap between the defined policies for work design and ergonomics between the country and company levels. Country specific policy in this domain may have been felt unnecessary since ergonomic design is based on international and well-known approaches and standards (i.e. ISO 11228). The stronger involvement of companies in this domain could also be attributed to the need to fit ergonomic and design standards to different workplace environment. For example, sectors like manufacturing, healthcare, and transportation often have specific ergonomic standards due to the physical nature of the work involved. Therefore, company level initiatives are the most effective and allow organizations to have more flexibility and customization options compared to country level policies. Companies can adapt guidelines based on their work environment and specific tasks. Our study revealed that different ergonomic solutions and working design practices are applied in studied countries to address ageing workers specific needs. For example, studies from Italy showed the importance of implementing the ergonomic solutions and several models have been applied and validated in companies. Boenzi *et al.* (2015) developed a case study concerning the implementation of age-related model to find the optimal job rotation schedules in work environments characterized by low load manual tasks. Peruzzini and Pellicciari (2017) developed a case study schedule the activities for aged workers exposed to the risk of repetitive tasks and the proposed model includes ergonomic risk assessment. Digiesi *et al.* (2018) designed and scheduled human-based assembly systems to balance of MSDs risk among workers with different age and skill and validated it in an Italian company. Botti *et al.* (2017) examined how to minimize the ergonomic risk associated with repetitive tasks by assigning appropriate tasks to the workers which better fit to their skills and capabilities. Furthermore, Katiraee *et al.* (2021a) developed a work-task categorization matrix considering workers’ differences in terms of expertise and perceived physical exertion. Their matrix helped workers to be involved individually in job assessment and resulted in less musculoskeletal disorder risk and worker’s fatigue. All the mentioned studies have considered workforce differences in terms of age in real case studies from industrial and manufacturing companies and consider work design and ergonomic factors in addressing aging workforce problems.

US companies are also seen to be attentive to ergonomic issues in workplace design. Offices often incorporate adjustable seats and keyboard trays to facilitate the use of ergonomically designed tools in manufacturing. Additionally, service workers’ tool undergo frequent assessments to ensure they meet ergonomic standards ( Nagarajan & Sixsmith, 2023). Brooks Brothers LIC Factory is a good example in this respect, implementing Job Restructuring and Physical Work Ergonomics, while Eneslow Pedorthic Enterprises Inc. employ ergonomic equipment in their workshops. ( Columbia research, 2023). More work is required to develop ergonomic design solutions for aging worker needs, such as steps to overcome common ailments like musculoskeletal disorders, and new ergonomic training programs.

### 5.3 Domain 3: Health management

Analysing this domain at the country/regional level, the European commission is seen to have developed several strategic frameworks for health and safety at work, which all EU countries, including France, Germany, Italy, and Slovenia, are obligated to implement in national laws or regulations. For example, as mentioned in Section 4.1 the “Strategic Framework on health and safety at work 2021–2027” was developed by OSHA to enhance the prevention of workplace accidents and illnesses, as well as to improve readiness for any potential future health crises. In New Zealand, health and safety guidelines are established by WorkSafe New Zealand. An overview of the Health of Older People Strategy developed by “Ministry and District Health Boards (DHBs)” is a strategy that targets people aged 65 and above. The integrated approach to service provision outlined in the document will be advantageous for older people with extensive and intricate requirements that span many service sectors. This strategy has multiple objectives: (1) Older people, along with their immediate and extended family can make well-informed choices. (2) The integration of policy and service planning is centred on the specific needs of older people. (3) Financial resources and operational assistance facilitate the availability of high- quality health and disability support services. The Ministry and DHBs must collaborate with other agencies to establish a framework that promotes more coherent health and disability support services for older people and to eliminate obstacles to their inclusion. (4) Appropriate, integrated health and disability support services to address the order workforce’s well-being. (5) Older people have timely access to primary and community health services. For health improvement and collaboration with health promotion programmes ( District Health Boards & Ministry of Health NZ, 2002).

Our data indicates that the health management domain attracts less attention at the company level. According to the Les Amis case study, there is no specific age limit for workers, and they are encouraged to continue working there (at Les Amis) even after 60 years of age. However, the lack of fitness of workers to work leads to four to five layoffs a year. The possibilities for increased flexibility in work practices remain limited, with the exception of reduced working hours and the ability for home aids to negotiate with clients the tasks to perform. The implementation of flexible working hours, along with annual working time accounts, is an additional element of the health management policy of the Sozial-Holding's corporation in Germany. Through the personalized working time models, allowing employees to plan their working hours flexibly and independently. In Dupont company in US, a health promotion campaign is set by promoting health, fitness, and aerobics program for older workers to make them more ready for demanding tasks.

### 5.4 Domain 4: Knowledge management

Knowledge management is a significant area of focus at the national level, especially in countries like Italy, Japan, and Slovenia (as shown in Figure 5). This field has also received considerable attention at the corporate level in countries including Canada, France, and Italy (referenced in Figure 6). In Italy, for instance, knowledge management emphasizes providing access to and encouraging participation in educational, training, and skill development programs. This approach is designed to facilitate employees' (re)entry and full participation in the labour market, particularly in high-quality jobs ( Council of European Union, 2012). Additionally, it's crucial for facilitating knowledge transfer from older to younger generations and for offering educational programs on active and healthy lifestyles, as well as promoting intergenerational solidarity and family support ( EU-OSHA, 2012). A prime example of effective practice is seen in the Italian company *API Raffineria di Ancona S.p.A*., which offers regular training to its employees every three months. This training covers technical/professional skills, safety, and environmental issues, and is available to all employees ( Naegele & Walker, 2006). Similarly, *Michelin* in Italy provides older employees with training, information, orientation, and counselling ( Naegele & Walker, 2006). In Slovenia, knowledge management efforts include similar access to education, training, and skill development. *Triglav Insurance Company*, for instance, provides employees with an online learning portal that is accessible anytime and anywhere. This was particularly beneficial during the coronavirus epidemic, offering webinars on stress management during self-isolation, working with virtual teams, and adapting to life post-coronavirus, with active participation from older employees ( Ramovš & Svetelšek, 2020). Another example of a successful Slovenian company is *Unika TTI* d.o.o. Here, the company's employment policy, as described by their director, follows a 'natural selection' principle. As an employee nears retirement, they gradually hand over their responsibilities to a younger colleague ( Ramovš & Svetelšek, 2020).

### 5.5 Domain 5: Intergenerational coexistence

Based on the AARP Global Employer Survey 2020, less than 6% of employers applied policies which are specifically designed to support multigenerational workforce ( OECD, 2020). However, a wide range of companies are eager to undertake additional efforts. Based on our data, the promotion of active ageing is a relative new area of intervention in all the countries analysed. The majority of initiatives aimed at promoting employment and retaining for older workers have been carried out at regional or provincial level, as part of ESF Regional Operational Programs ( Barabaschi, 2015). The solidarity agreement promotes work sharing by facilitating and encouraging the transformation of full-time contracts for workers over the age of 55 into part-time contracts. Additionally, it seeks to create part-time employment opportunities for unemployed under the age of 25 or 30, if they have a university degree.

In Slovenia, many studies were carried out (both at companies and national level), focusing on age management and intergenerational coexistence (e.g., Erjavšek, 2005; Mejaš *et al.*, 2014; Rožman *et al.*, 2020; Tomšič, 2010; Veingerl Čič & Šarotar Žižek, 2017). The research done by Veingerl Čič and Šarotar Žižek (2017) revealed that several authors have noticed significant and relevant generational differences among employees in workplaces. Veingerl Čič and Šarotar Žižek (2017) developed a model that identifies key areas influenced by management in the implementation of intergenerational interaction. The Slovenian project ‘We All Win When We Work Together’, seeks to enhance awareness across all age groups regarding the importance of teamwork and lifelong learning. However, generalised offerings are not without issues. A German sensor intelligence business provided training sessions done in several age groups to prevent age-based discrimination. However, IT training proved to be an exception in this regard, since older and younger employees have different learning rates for new media. Consequently, older employees require more time to familiarize themselves with the content. IT training is now carried out in groups with employees of a similar age in order to prevent conflict and stress. The company values its senior employees, as seen by the fact that they are considered mentors for the younger employees, especially when it comes to sales and trainee promotion. Similarly, IBM has a strategy for intergenerational diversity which focuses on attracting, motivating, and retaining the most talented individuals in the industry. IBM created a comprehensive approach to personal growth with a particular focus on older employees, consisting of three phases: (1) attracting and retaining employees, (2) policies for the pre-retirement stage – identifying stage policies. (3) strategies for attracting, motivating, and retaining post-retirement individuals and older employees.

### 5.6 Domain 6: Retirement pathways

Figure 5 and Figure 6 show that all countries have considered retirement pathways at both country and company levels of intervention. Several measures are applied to ease and facilitate retirement as an incremental, gradual process. These measures predominantly refer to flexible work arrangement and skill training and abilities improvement to retain older workers. On the other hand, there appears a lack of other possible measures such as work-life balance, personal life support, building senior workers’ entrepreneurship, self-employment, and career development, and transition solutions to retirement. EU and Canada evidence relative resistance from the employer side to apply such measures. We note the most advanced system of inclusion of older workers are in USA and Japan, where the retirement pathways are also the least regulated and mandatory by laws. Japan represents the benchmark of the retirement pathways domain. Japan has the highest rate of employment among older workers within the OECD, with 50% of those aged 65–69 employed in 2020, which is significantly higher than the OECD average of 23%. Additionally, individuals in Japan can opt for late retirement between the ages of 65 and 70, receiving a 0.7% increase in benefits for each month they delay retirement past the age of 65. Since 2004, workers over 65 have been able to combine employment income with their pension, provided their total income doesn't surpass JPY 470,000. If earnings exceed this threshold, the full pension is still paid, but half of the amount above this limit is deducted from the pension related to earnings. Furthermore, individuals over 70 are exempt from making pension contributions ( OECD, 2021c). Japan also has the highest average retirement age in the labour market, with women retiring at an average age of 66.2 and men at 68.2. However, the regulations governing work and pension may deter some individuals from continuing to work ( OECD, 2021d). Japan also offers Silver Human Resource Centers, a network of employment centres, that help unemployed older workers to find jobs and offer comprehensive public welfare programs for older citizens who desire to be active after retirement ( Flynn *et al.*, 2014). Another good case is the Public Employment Service in Japan, called Hello Work, which provides Japanese with different services encompassing consulting, guidance and job placement for 55+ and 65+ years old, who search for jobs ( OECD, 2018b). The Japanese government promotes flexible work practices and requires employers to design such practices, although there are only limited penalties for those organizations, which do not comply. The Japanese government amended the Labor Standard Law (1947) as early as 1987, asking organizations to design flexible working hours, including flexitime and compressed work hours. The adoption for employers was predominantly voluntary ( Flynn *et al.*, 2014).

According to Figure 6, the most active countries in terms of company best practices in this domain are Japan and US. The Mirai Industry Co in Japan does not promote overtime work, enabling employees work-life balance. Workers who do not perform overtime work are perceived as good employees. The savings from avoiding overtime is given to employees as a subsidy for lunch money. Every employee is hired on a regular contract (not temporary or part-time) with retirement at 70 years of age. Their salaries are among the top in Gifu Prefecture, where they operate ( Baudrand *et al.*, 2018). At Nojima Co. (electronics retailer) they enable employment to the people age 70+. Their policy regarding the age of employment changed in 2020, as they raised it to 80 years, and from 2021, they abolished the age limit of seniors. Rather, they decided whether they will allow the employment on a case-by-case basis, depending on the candidate’s health condition. At the end of January 2022, they had 25 seniors aged 70+ working ( Kyodo, 2022). At Tokyu Community Corp. in Tokyo they contract senior workers or employ them as part-timers until 77 years old. Almost half of their 11,322 employees in March 2021 were 65+, and 2,320 were 70+. Several of those workers retired from other companies and industries and work as maintenance staff. They offer extensive training programmes and a reward of ¥5,000+ for seniors who acquire real estate-related qualifications. Their older employees, who work shorter hours per week and earn lower income, are allowed to have a side job from the other industry ( Kyodo, 2022).

Corporate America (US) has also been active in managing the aging workers. Pitney Bowes, a major technology corporation based in Connecticut with 14,000 employees, provides an educational grant known as the Retirement Education Assistance Program. This program, offering up to $3,000, enhances the ability of older employees to manage new technologies ( Collins & Casey, 2017). Similarly, Procter & Gamble and Siemens have implemented reverse mentoring programs in which younger staff educate managers and executives, often in the mid-career or older age groups, about contemporary technologies ( Collins & Casey, 2017). RWE Power has established training programs aimed at helping older employees to refresh their skills and capitalize on their experience. Such initiatives have yielded benefits in terms of enhanced skills among older workers, increased productivity, and heightened motivation through performance incentives ( Strack *et al.*, 2008). Eneslow Pedorthic Enterprises Inc. utilizes strategies like gradual retirement and flexible scheduling to support aging workers ( Columbia Research, 2023). Betz Laboratories actively hires older workers to lower staff turnover ( Auer & Fortuny, 2000). Xerox has introduced the Flexible Work Initiatives Senior Employee Program, which offers reduced hours and responsibilities for employees over 50 ( Auer & Fortuny, 2000). Days Inn, operating in the hospitality sector, has also seen significant benefits from employing older workers. Previously struggling with high turnover rates at its reservation centres, the company chose to hire individuals aged 55 and older to replace 30% of its departing staff. This change led to a turnover and absenteeism rate among older employees of less than one percent, and a 40 percent reduction in costs related to turnover and training, alongside increased customer satisfaction. Days Inn works to retain these senior employees by offering flexible work schedules, career advancement opportunities, a program benefitting grandchildren, and various incentives and bonuses ( Auer & Fortuny, 2000).

## 6. Conclusive remarks

This study develops a roadmap ( Figure 3) to guide and support age-inclusive workforce management policy and practice. The road map develops and describes 6 main domains of age-oriented action at 3 different levels of intervention: international level, country level and company level. The authors have collected and analysed a total of 12 international policies, 74 country level policies and 92 company best cases coming from the 8 countries involved in this project: 4 European countries (France, Germany, Italy, and Slovenia) and 4 Worldwide countries (Canada, Japan, New Zealand, and USA).

The analysis reveals that ageing workforce management is currently a research topic of emerging interest in many different countries and company sectors. Age-oriented policies and best practices are being increasingly designed and implemented albeit at different speeds and foci among the 8 analysed countries. A common element among all the countries analysed is the general awareness that ageing workers’ competencies and skills are extremely valuable for many industries. Companies are keen to introduce guidelines and flexible working conditions that would help retain ageing workers (or their IP – intellectual property) as long as practically possible. However, new initiatives and best practices are required at all levels to help achieve this goal. In particular, the following aspects requires a further investigation both by governmental institutions and both by private companies:

*1.* According to the collected data ( Figure 5 and Figure 6), there is an important observation regarding the difference in attention given to aging workforce policies at the country level compared to the company level. It’s true that national policies often focus on broader societal goals and may not directly impact on company’s profitability in the short term. However, there are several reasons why companies should still consider adopting an implementing such policies:

- *Long-term sustainability:* while aging workforce policies may not yield immediate profits, they contribute to the long-term sustainability of a company. Implementing age-friendly policies can help companies retain valuable aging workforces.
- *Reputation and Brand Image:* Companies that are known for their commitment to diversity, inclusion, and supporting an aging workforce tend to have a positive reputation. A good reputation can attract top talent and customers who appreciate socially responsible practices.
- *Workplace Morale and Productivity:* A healthy work environment that values all employees, regardless of age, can lead to higher morale and increased productivity. Employees who feel respected and supported are often more motivated and engaged in their work.
- *Legal and Regulatory Compliance*: In some countries, there are legal requirements related to age discrimination and the treatment of older workers. Failure to comply with these laws can result in legal troubles and financial penalties.

*2*. Data ( Figure 5 and Figure 6) indicates that, work design and ergonomic policy is less encountered at the country level as compared to the company level, highlighting an interesting dynamic in aging workforce management. There could be several reasons:

- *Tailored solutions:* Each company's manufacturing processes, work environments, and workforce demographics can vary significantly. Therefore, a one-size-fits-all country policy may not adequately address the specific ergonomic and work design needs of individual companies.
- *Specific Requirements:* Different industries within manufacturing may have unique ergonomic challenges. For example, the ergonomic needs of an assembly line in the automotive sector may differ from those in a food processing plant. Customization allows companies to address industry-specific challenges effectively.
- *Workforce diversity:* Companies may have employees of varying ages, physical abilities, and health conditions. Customization enables companies to design workspaces and processes that accommodate the specific needs of their diverse workforce.
- *Workforce engagement:* Involving employees in the customization of work design and ergonomic policies can lead to higher engagement and satisfaction. Employees often have valuable insights into their own ergonomic needs and can provide input on practical solutions.

However, it’s important to strike a balance between customization at the company level and having a broader framework at the national level. Hence, while customization of work design and ergonomic policies at the company level is essential, there remains a role for country guidelines and oversight to ensure worker safety, share best practices, and provide a foundational framework for addressing the challenges posed by an aging workforce.

*3. Promote experience and expertise*, *mentorship, and knowledge transfer:* provide new guidance and support methods to manage the knowledge transfer in order to ensure that critical institutional knowledge is retained within the organization, even as older workers retire. Therefore, providing collaborative work environment can help companies to foster a culture of collaboration where employees of all ages are encouraged to share knowledge and ideas. Based on the collected data, here, there are some ways to encourage collaboration among employees of all ages:

- *Diversity and inclusion initiatives:* ensure that all employees, regardless of age feel included and valued for their contribution and knowledge. This diversity can lead to the exchange of fresh perspectives and experiences.
- *Knowledge-sharing platforms:* invest in digital collaboration tools and platforms that enable employees to share documents, best practices, and project updates easily.
- *Feedback and continuous improvement:* solicit feedback from employees regarding collaboration efforts and knowledge transfer processes. Use this feedback to continuously improve collaboration strategies within the organization.
- *Clear documentation:* encourage employees to document their knowledge and expertise in easily accessible formats. Also emphasize the importance of creating clear and well-organized documentation for future reference.

*4. Promote diversity and inclusion in manufacturing and logistic sectors:* a multi-generational workforce brings together individuals with different perspectives, ideas, and work styles. This diversity can lead to enhanced creativity, innovation, and problem-solving within organizations, also in the manufacturing and logistics sectors in which many jobs are still not inclusive.

*5. Promote the Extension of career lifespan:* by creating appropriate work environments and implementing age-friendly policies, organizations can support aging workers in remaining productive, active and engaged for longer periods.

*6. Introduce economic benefits for extending the career lifespan:* an aging workforce can have positive economic impacts. By keeping experienced workers in the labour market, organizations can avoid the loss of valuable skills and knowledge. This can lead to cost savings in terms of recruitment, training, and knowledge acquisition for the organization. Additionally, older workers who continue to work contribute to the economy by paying taxes and generating income, which can have a positive effect on society. Governments can accelerate this process through effective policy and laws.

## Ethics and consent

Ethical approval and consent were not required.

## Data Availability

All data about the applied policies and strategies for aging workforces are taken from websites, previous studies, and articles and collected by the involved partners in this project. All collected data are summarized in the file name Tables_Appendix including three tables name (A1, A2 and A3) with their recourses and citations in the repository below: Research Data Unipd: A new roadmap for an age-inclusive workforce management practice and an international policies comparison. https://doi.org/10.25430/researchdata.cab.unipd.it.00001220 ( Katiraee *et al.*, 2024). Data are available under the terms of the Creative Commons Attribution 4.0 International license (CC-BY 4.0).
