# Supplementary material for: A new roadmap for an age-inclusive workforce management practice and an international policies comparison
Source: Open Res Eur. 2024 Jun 24;4:85. Originally published 2024 Apr 24. [Version 2] doi: 10.12688/openreseurope.17159.2 (PMC11200061; doi:10.12688/openreseurope.17159.2)
Supplement: Peer review history

# Reviewer report: Reviewer response for version 2

**DOI:** 10.21956/openreseurope.19524.r40486

**Published:** 2024-07-03

**Authors:** Bernadeta Gostautaite

**Affiliations:** 1 Vilnius University, Vilnius, Lithuania

This study presents a research-based roadmap to support age-inclusive workforce management. The roadmap integrates published literature, best practices, and international policies, providing a comparison of age-inclusive practices at international, country, and company levels, including data collected from eight countries. The research is particularly relevant given that the global workforce is aging rapidly, prompting governments and companies to address age-related challenges in workforce management, while developing age-friendly working environments to enhance inclusion and diversity among aging workers has become a key priority.

 The authors have successfully curated a comprehensive review of numerous studies spanning various fields of research, compiling exemplary practices from different countries and organizations. This compilation is of significant value, and I commend the authors for their excellent work.

 Additionally, I would like to offer some recommendations that could enhance the paper further.

- Could you please provide more detailed explanations on the current theories concerning “ageing workers” or consider omitting this introduction altogether in the paper? I believe that your theoretical framework should address age management in organizations more so than the process of aging at an individual level.
- I recommend revising the title of the “2.3 Workforce diversity” section to reflect its focus on age-related changes throughout a person's career rather than broader diversity concerns.
- While I value the quick summary of current models like the Later Life Work Index (LLWI), I recommend highlighting how the new framework diverges from these and what unique contributions it offers.
- The segment discussing how the 6 domains should be prioritized according to the particular requirements, objectives, and nature of businesses is crucial, and I recommend detailing it further. For example, could you define "cost," "effort," and "time" with greater specificity?
- I suggest removing reviews from the list of initiatives, for instance, “The 2013 evaluation of the European 2007–2012 strategy,” given that an assessment report doesn't result in a novel initiative or policy. If feasible, it would be beneficial to include details on the impact of each initiative derived from an evaluation report.
- Thanks for gathering information on various initiatives. Could you add the initiative year in Tables A2 and A3 for a snapshot of their progression over time?
- Consider including a detailed explanation of your data-gathering techniques and sampling processes, like the methods used to find relevant initiatives.  
- I suggest you elaborate on the most significant remaining gaps and propose future directions for research and policymaking in the field of age management.

Congratulations on your excellent and invaluable contribution.

If applicable, is the statistical analysis and its interpretation appropriate?

Not applicable

Is the study design appropriate and is the work technically sound?

Yes

Is the work clearly and accurately presented and does it engage with the current literature?

Partly

Are the conclusions drawn adequately supported by the results?

Yes

Are sufficient details of methods and analysis provided to allow replication by others?

Partly

Are all the source data and materials underlying the results available?

Yes

Reviewer Expertise:

aging at work, adaptation to new technology, age management in organizations, career across lifespan

I confirm that I have read this submission and believe that I have an appropriate level of expertise to confirm that it is of an acceptable scientific standard, however I have significant reservations, as outlined above.

# Reviewer report: Reviewer response for version 2

**DOI:** 10.21956/openreseurope.19524.r41602

**Published:** 2024-06-27

**Authors:** Francesco Lolli

**Affiliations:** 1 University of Modena and Reggio Emilia, Reggio Emilia, Emilia-Romagna, Italy

I have no further comments.

If applicable, is the statistical analysis and its interpretation appropriate?

Not applicable

Is the study design appropriate and is the work technically sound?

Yes

Is the work clearly and accurately presented and does it engage with the current literature?

Yes

Are the conclusions drawn adequately supported by the results?

Yes

Are sufficient details of methods and analysis provided to allow replication by others?

Yes

Are all the source data and materials underlying the results available?

Yes

Reviewer Expertise:

Risk assessment and safety management

I confirm that I have read this submission and believe that I have an appropriate level of expertise to confirm that it is of an acceptable scientific standard.

# Reviewer report: Reviewer response for version 2

**DOI:** 10.21956/openreseurope.19524.r41603

**Published:** 2024-06-25

**Authors:** Seyyed Ehsan HASHEMI-PETROODI

**Affiliations:** 1 KEDGE Business School, Bordeaux, France

I appreciate the work of authors for modifications in the new version and their responses. The work can be accepted, it is a great job and very interesting work!

If applicable, is the statistical analysis and its interpretation appropriate?

Yes

Is the study design appropriate and is the work technically sound?

Yes

Is the work clearly and accurately presented and does it engage with the current literature?

Yes

Are the conclusions drawn adequately supported by the results?

Yes

Are sufficient details of methods and analysis provided to allow replication by others?

Yes

Are all the source data and materials underlying the results available?

Yes

Reviewer Expertise:

Operations Research, Manufacturing system design, Assembly line balancing, Workforce planning, Robust optimization

I confirm that I have read this submission and believe that I have an appropriate level of expertise to confirm that it is of an acceptable scientific standard.

# Reviewer report: Reviewer response for version 1

**DOI:** 10.21956/openreseurope.18545.r40483

**Published:** 2024-06-07

**Authors:** Seyyed Ehsan HASHEMI-PETROODI

**Affiliations:** 1 KEDGE Business School, Bordeaux, France

The paper proposes a roadmap for age-inclusive workforce management. The literature is reviewed, and the authors compare and discuss age-inclusive management practices and policies based on six domain actions (organizational culture and leadership, work design and ergonomics, health management, knowledge management, intergenerational coexistence, and retirement pathways) at three different levels of intervention: international, country, and company. Data collection and analysis were conducted in eight countries: Canada, France, Germany, Italy, Japan, New Zealand, Slovenia, and the USA. Finally, several aspects are proposed for future research in the conclusion.

 The work is interesting and well-structured. The ideas and discussions presented in the paper are interesting, and the work is sound. There are some remarks/questions to improve the work as follows:

- The main contributions/findings of the paper should be highlighted at the beginning of the work (perhaps in the introduction or end of literature).
- The reference for the information given in Figure 1 should be noted and discussed more thoroughly in the text.
- The authors should justify the three sub-sections proposed and explain the link between them. A short description could clarify what will be discussed in the three sub-sections.
- I liked the idea of describing the link between each of the six domains as done at the end of sections 3.3 and 3.4. However, similar discussions are lacking for other domains. Authors can address this. Additionally, it should be clarified how the link (or position) of the six domains aligns with the three proposed levels. The analysis could be conducted this way, and perhaps Figure 3 can be improved (or maybe a new figure created) to show the position/link of domains and levels.
- It is good to see how the 92 companies studied in section 4.3 differ. What are their major sectors? An analysis could also be done for the different types of companies studied per country.
- Sections 5 and 6 are well-presented and include some discussions and future research focuses. However, in my opinion, they deserve more discussion and propositions regarding methodological and scientific contributions for the future. Several aspects are presented, but perhaps the authors can elaborate more on the solutions and methodological contributions to address these aspects.
- Including some tables and figures to present the main messages and comparisons, especially in sections 3, 4, and 5, could be a good idea. Such presentations would be more engaging than text alone in this type of research. Some minor remarks are: 1) In the first sentence of section 4, is it “previous paragraph”? 2) The authors should check the use of abbreviations. Sometimes they are defined multiple times, e.g., MSD.

If applicable, is the statistical analysis and its interpretation appropriate?

Yes

Is the study design appropriate and is the work technically sound?

Yes

Is the work clearly and accurately presented and does it engage with the current literature?

Yes

Are the conclusions drawn adequately supported by the results?

Yes

Are sufficient details of methods and analysis provided to allow replication by others?

Yes

Are all the source data and materials underlying the results available?

Yes

Reviewer Expertise:

Operations Research, Manufacturing system design, Assembly line balancing, Workforce planning, Robust optimization

I confirm that I have read this submission and believe that I have an appropriate level of expertise to confirm that it is of an acceptable scientific standard, however I have significant reservations, as outlined above.

# Response

**Published:** 2024-06-11

**Authors:** Niloofar Katiraee

**Affiliations:** Management and Engineering, University of Padua, Vicenza, Veneto, Italy

*We are grateful for the valuable revisions provided by the reviewer, which have contributed to the enhancement of our work. We have tried to incorporate the suggested revisions and have provided responses to the reviewer's feedback. *

- In fact, the main contribution of this work is to develop a methodological roadmap based on data obtained from various global partners. This roadmap aims to enhance the management methods for aging workers in workplaces, supporting their inclusion and retention by fostering age-inclusive workforce management practices. And we tried to highlight it at the end of the introduction.
- In section 2.1, we explained completely about the Figure 1 that what information it includes.
- In section 2, we added information about the three sub-sections of the literature review.
- We agree with reviewer about the same connection between other domains, however we highlighted these connections more prominently for domains that have significant overlap such as domain 2 and domain 3. In this version we also added the connection between domain 4 and 6.  

Regarding the position of the six domains, we added more information in section 3, that “ *the mentioned domains are generally equally important, but their level of importance can vary from one country to another depending on the country's perspective and policies, or from one company to another, based on the nature of the tasks involved.*” While all domains are equally crucial, the culture of each organization (Domain 1) serves as the foundational step. Subsequently, addressing ergonomic and health concerns of workers (Domain 2 and 3), facilitating knowledge transfer and intergenerational coexistence (Domain 4 and 5), and finally, managing retirement pathways are essential steps (Domain 6). And all these domains are investigated at three levels (up to down): International, Country and company. Therefore, in the proposed figure, we've maintained equal size and position for the domains by placing the three levels at the center, reflecting their equal importance across different contexts.

- Acknowledging the importance of the company sector is indeed crucial. While our data collection encompassed various sectors and resources, our focus remained on the policies and strategies implemented by these entities. Nevertheless, we have provided all references and links in the appendix, enabling readers to delve deeper into the specifics of each company's approach.
- This paper presents a final roadmap derived from a thorough investigation of previous studies, an extensive literature review, and data collection. By implementing this roadmap across three levels, we identified a gap in used management methods for ageing workforces, elaborated on in Section 6 as a subject for future investigation. Our proposed solution is the new roadmap tailored to address the challenges posed by ageing workforces. Hence, we maintained section 6 to focus only on required aspects for future consideration.
- Sections 3, 4, and 5 delve into the significance of each domain through discussions of previous studies. Due to this approach, there isn't a specific categorization suitable for tables or figures. However, we have included Figures 6 and 7, as well as tables in the appendix, illustrating how the domains have been considered by each partner at the country and company levels. Additionally, in this version, we've provided more detailed explanations indicating the main focus of each domain in Section 3 to provide clarity on their importance.
- **Minor revisions addressed**: We appreciate you pointing these out, and we have made the necessary corrections.

We extend our appreciation once again to the reviewer for their invaluable insights. We believe that the revisions made in this new version, along with our detailed responses to the reviewer's comments, sufficiently address any concerns and ensure the satisfaction of the reviewer.

# Reviewer report: Reviewer response for version 1

**DOI:** 10.21956/openreseurope.18545.r40484

**Published:** 2024-06-04

**Authors:** Francesco Lolli

**Affiliations:** 1 University of Modena and Reggio Emilia, Reggio Emilia, Emilia-Romagna, Italy

The manuscript provides a wide picture of policies and best practices adopted in eight countries to address age-related workforce management. In particular, three levels of intervention (International, country and company) and six action domains are investigated to develop a roadmap facing challenges and opportunities that an aging workforce imposes.

 I think that this is an excellent piece of work related to a very current and relevant topic for the management of modern businesses towards more inclusive workplaces, which are a key element of the long-term sustainability for companies.

If applicable, is the statistical analysis and its interpretation appropriate?

Not applicable

Is the study design appropriate and is the work technically sound?

Yes

Is the work clearly and accurately presented and does it engage with the current literature?

Yes

Are the conclusions drawn adequately supported by the results?

Yes

Are sufficient details of methods and analysis provided to allow replication by others?

Yes

Are all the source data and materials underlying the results available?

Yes

Reviewer Expertise:

Risk assessment and safety management

I confirm that I have read this submission and believe that I have an appropriate level of expertise to confirm that it is of an acceptable scientific standard.
